# The Effects of a Digital Game Simulator versus a Traditional Intervention on Paramedics’ Neonatal Resuscitation Performance

**DOI:** 10.3390/children11020174

**Published:** 2024-01-30

**Authors:** Maria Cutumisu, Georg M. Schmölzer

**Affiliations:** 1 Department of Educational and Counselling Psychology, Faculty of Education, McGill University, Montreal, QC H3A 1Y2, Canada; maria.cutumisu@mcgill.ca; 2 Centre for the Studies of Asphyxia and Resuscitation, Neonatal Research Unit, Royal Alexandra Hospital, Edmonton, AB T5H 3V9, Canada

**Keywords:** neonatal resuscitation, game, simulation, digital, paramedics, performance

## Abstract

Neonatal resuscitation is a skill set that comprises procedures, assessment, decision-making, communication, and teamwork. It is used in an emergency situation in the delivery room with the aim of supporting newborn infants who are not able to begin breathing on their own. Thus, healthcare providers need to refresh their neonatal resuscitation skills periodically, according to the Neonatal Resuscitation Program, to ensure that they can react quickly and effectively in emergency situations. The RETAIN digital game simulator was designed to enable healthcare providers to practice their neonatal resuscitation skills. To evaluate the effectiveness of this game in a laboratory setting, a randomized control trial sampled 42 paramedics who completed a pre-test, were randomly assigned to watch a traditional lecture video on the neonatal resuscitation procedure or to play a novel digital game simulation on the same topic, and then completed a following test. A two-way mixed ANOVA revealed a statistically significant improvement in paramedics’ neonatal resuscitation performance over time, which did not differ between conditions. Thus, digital games can provide an enjoyable alternative to traditional practices in refreshing neonatal resuscitation knowledge.

## 1. Introduction

Approximately 10% of neonates require various degrees of respiratory assistance at birth and fewer than 1% infants require extensive resuscitation [1]. The process of neonatal resuscitation draws on technical, communication, and teamwork skills. A breakdown in any of the parts of the neonatal resuscitation process can result in distraction, decision making deficiencies, medical errors, poor patient outcomes, and it can put infants at risk of irreversible organ injury and even death. Indeed, approximately 66% of neonatal deaths during resuscitation are caused by a breakdown in decision making, communication, and teamwork rather than solely in technical skills (e.g., mask ventilation; [2,3]).

Therefore, healthcare providers (HCPs) must possess the knowledge and skills to perform the steps of the neonatal resuscitation algorithm standardized by the Neonatal Resuscitation Program [4,5,6]. The neonatal resuscitation algorithm outlines the current approach for managing newborn infants at birth. However, current training modalities tend to focus exclusively on individual and technical skills (e.g., mask ventilation), and are usually carried out in specialized simulation centers [7]. These centers and their resources are expensive to maintain and difficult to access around the clock. Such traditional neonatal resuscitation training methods are also sensitive to events such as the recent COVID-19 pandemic, when many in-person simulation sessions have been canceled due to staff shortages and physical distancing rules. These situations have caused losses in training opportunities, which ultimately can influence patient outcomes. Moreover, it has been reported that HCPs find it difficult to strictly adhere to resuscitation guidelines [8].

Thus, we developed the RETAIN (REsuscitation TrAINing) digital game [9,10] as an online simulator that mimics the real-life neonatal scenarios occurring in a delivery room to provide HCPs with training opportunities that are more accessible to them at any day and time, without having to make an appointment for in-person training or refresher sessions.

Games have been generally defined as structured or organized play [11]. Digital games constitute games developed using computer technology. They have been used extensively in educational research as testbeds for experimentation with different instructional methods to ascertain their impact on student outcomes (e.g., motivation, learning, etc.). Their use in academic research has been accelerated by James Paul Gee’s [12] work, which emphasized several design principles that make games important vehicles for learning. Valerie Shute’s [13] work on stealth assessments has also emphasized the role of games as valid and reliable assessment tools designed to measure 21st century skills [14]. Much of the appeal for digital games is derived from the motivation that they can instill in the players. In their Self-Determination Theory, Deci and Ryan [15] posed that in-game perceived autonomy, competence, and relatedness foster intrinsic motivation from which enjoyment is often derived.

More recently, digital games have been adopted in medical training and research, where computer technology has facilitated the creation of realistic simulation environments where learners can explore concepts and behaviors that are situated in the learning context (i.e., knowledge domain). Specifically, simulations based on games were developed to reap the benefits of games while setting themselves apart from games, as simulations are created by design to represent a real-world situation or system [16]. This type of situated learning is one of the themes of the Cognitive Apprenticeship Model [17], which aims to teach the procedures (i.e., knowledge, skills, behaviors, and attitudes) that experts employ when solving complex real-world problems. For example, BioWorld is an instance of the cognitive apprenticeship framework that simulates a medical environment [18,19,20]. In BioWorld, players assume the role of a medical doctor by reading a patient’s case and formulating hypotheses to solve the problem presented in a certain scenario. They can revise their hypotheses when new data become available to them.

A recent review of simulation-based training methods called for a prioritization of the integration of simulation systems into clinical environments, with the goal of improving patient safety and outcomes [7]. The present study is a first step in evaluating the efficacy of such a simulation training system based on a digital game. This experimental study aimed to ascertain participants’ perceptions of the RETAIN game and to determine whether the effects of the two forms of neonatal resuscitation instruction, the traditional method (lecture video) and the digital game, on participants’ performance on neonatal resuscitation scenarios are different over time. Thus, the present study poses the following research questions: (1) Do the two instructional groups differentially change their paramedics’ neonatal resuscitation performance from a pre-test to a post-test? (2) Do participants perceive the digital game positively?

## 2. Materials and Methods

### 2.1. Participants and Procedure

This experimental study took place in a laboratory setting and sampled 42 paramedics from the Edmonton Zone Emergency ambulance service. There were no exclusion criteria for the participants. Most participants completed a diploma, certificate, or another professional program (40 or 95.2%), a bachelor’s degree (1 or 2.4%), or an after degree (1 or 2.4%). They reported an average of M = 40.07 months (SD = 41.69) since their last NRP course training. The participants completed a consent form, according to the University of Alberta Ethics Board protocol Pro00117010.

First, all participants completed a survey that included demographic and background information questions (i.e., age, gender, professional designation, etc.).

Second, all participants completed a pre-test. The scenario used in this test included the provision of resuscitative care to a near-term infant with a focus on the initial steps of resuscitation (i.e., stimulation, temperature management) and respiratory support. All participants performed a simulation of a neonatal emergency using a manikin.

Third, participants were randomly assigned to a control (watched a lecture video on the neonatal resuscitation procedure) or a treatment (played a digital game simulation on the same topic) condition (i.e., group). Randomization was conducted using a computer-generated randomization table. The materials for the participants in the control group consisted of a 20 min video lecture based on the 8th Edition of the Neonatal Resuscitation Program [4]. The lecture was designed by the research team, standardized for all of the participants in the control group, and tailored to the paramedics. The materials for the participants in the treatment group consisted of the simulated tutorial and game scenario of the RETAIN digital game. Participants played the RETAIN digital simulator, solving scenarios with varying degrees of difficulty. Participants from both conditions spent similar amounts of time on the activities (i.e., approximately thirty minutes).

Finally, all participants completed a post-test. The scenario used in this post-test was similar in format and difficulty to the one used in the pre-test. The assessors of the pre-test and post-test were not aware of the design of this experimental study. Additionally, the participants who played the RETAIN digital game also answered some items regarding this environment (e.g., enjoyment).

More information about the paramedics who participated in this experimental study can be found in Table 1.

### 2.2. Measurement Instrument: The RETAIN Digital Game

We have built a digital game, RETAIN, which simulates neonatal resuscitation scenarios. The purpose of this digital game is twofold: (1) to train new healthcare practitioners on how to carry out the official neonatal resuscitation program (NRP) algorithm and (2) to allow experienced practitioners to refresh their skills by practicing the algorithm. Importantly, this digital game also enables healthcare practitioners to practice the neonatal resuscitation algorithm anytime and anywhere, in a safe environment where they could attempt many procedures without the risk of harming the infant. In RETAIN, the player (i.e., a clinical care provider) takes on the role of a healthcare practitioner performing neonatal resuscitation. The RETAIN digital game includes a tutorial and 50 scenarios.

The tutorial session introduces the players to the simulation interface and walks them through the neonatal resuscitation procedure including drying the baby, assessing the baby’s breathing, assessing the baby’s color for cyanosis (blue or pink), applying suction to the mouth and nose to clear the airway, adjusting the head position to improve airway function, applying a bag valve mask to assist with breathing, applying chest compressions, endotracheal intubation, and administration of epinephrine. The tutorial covers the order of application for these interventions as well as the conditions under which each should be applied.

Upon completing the tutorial, the player navigates through different resuscitation scenarios. By playing through the game scenarios, the player assesses and provides the appropriate care for a simulated neonate (i.e., newborn baby) experiencing breathing difficulties at birth. In the clinical case scenarios, the player experiences different delivery room scenarios and performs various steps of the neonatal resuscitation process (e.g., suction, and adjustment of the head position, assisted breathing using the bag and mask, chest compressions or endotracheal intubation and administration of epinephrine).

### 2.3. Measures

This experimental study has included the following measures.

*Condition*. This between-subjects factor represents the instructional method used for training the participants. It takes two values: 1 (i.e., control: video of a lecture on the neonatal resuscitation algorithm) and 2 (i.e., digital game based on the neonatal resuscitation algorithm).

*Time*. This within-subjects factor represents the two time points when data about participant’s performance on the neonatal scenarios were collected: before (pre-test) and after (post-test) the instructional intervention.

*Performance*. This dependent variable represents a participant’s neonatal resuscitation knowledge on the various scenarios. It was collected at two time points: before (via the pre-test) and after (via the post-test, which was identical to the pre-test) the instructional intervention, respectively. It was coded with a 1 for each correct step and a 0 for each incorrect step. Thus, it ranged from 0 to 14.

## 3. Results

All the analyses were conducted using R version 4.3.2 [21].

### 3.1. Do the Two Instructional Groups Change Differentially Their Paramedics’ Neonatal Resuscitation Performance from a Pre-Test to a Post-Test?

A two-way mixed analysis of variance (ANOVA) was conducted to answer this research question and to determine whether any change in the participants’ performance of neonatal resuscitation scenarios was the result of the interaction between the type of treatment (i.e., the lecture video or the digital game) and time. The data set was screened for outliers and the assumptions for this analysis were explored. Descriptive statistics per group of the variables used in this analysis are shown in Table 2.

First, normality tests were conducted. The results showed that the performance scores of the participants were normally distributed, with a marginal *p*-value for the pre-test in the group that received the traditional method instructions, as shown in Table 3. Nevertheless, Appendix A includes the robust two-way mixed ANOVA to ensure that the results are consistent when correcting for the non-normality of the post-test scores in the traditional method group.

Moreover, Figure A1 from Appendix A shows that, in the QQ plot for each cell of design, all the points fall approximately along the reference line, so we can assume normality.

Second, descriptive analyses revealed no outliers; thus, all data points collected were included in the analyses. Third, Levene’s test was not significant [*F*(1, 40) = 0.02, *p* = 0.89], so the homogeneity of variance assumption was met. Finally, Mauchly’s sphericity test was not generated, as there were only two repeated measurement levels.

**Figure 1:**
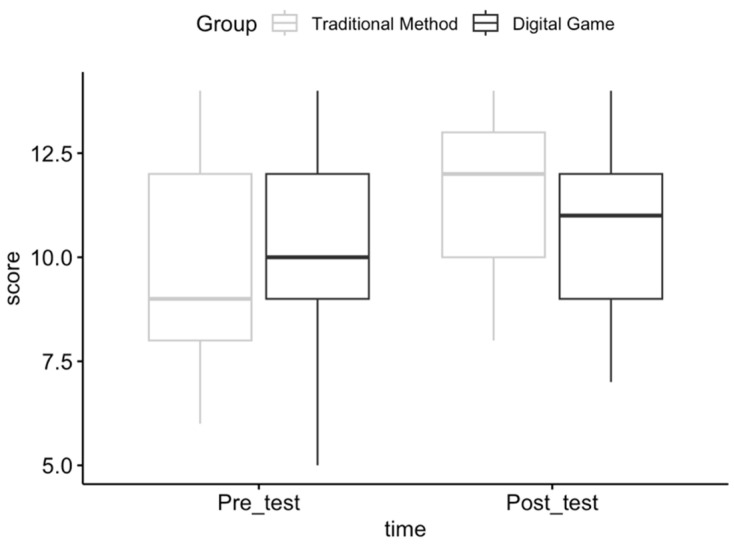
Box plot of participant performance by condition and time point.

The results of a two-way mixed ANOVA showed that there was no interaction between time and condition predicting participants’ neonatal resuscitation performance, as also seen in Figure 1.

However, there was a statistically significantly large main effect of time [*F*(1, 40) = 15.86, *p* < 0.001, *η_p_*^2^ = 0.28] on the neonatal resuscitation performance, as shown in Table 4.

To explore the single effect of time, a paired *t*-test for the time variable was conducted using the Bonferroni correction, ignoring the group. The results showed a statistically significant increase in performance [*t*(41) = −3.85, *p* < 0.001] from the pre-test (*n* = 42, M = 9.88, SD = 2.26) to the post-test (*n* = 42, M = 11.095, SD = 1.885).

### 3.2. Do Game Players Perceive the Digital Game Positively?

Descriptive statistics were conducted for the participants in the digital game group to answer this question. A total of six 5-point Likert scale items from the post-survey administered to the participants who played the RETAIN game are depicted in Table 5.

**Figure 2:**
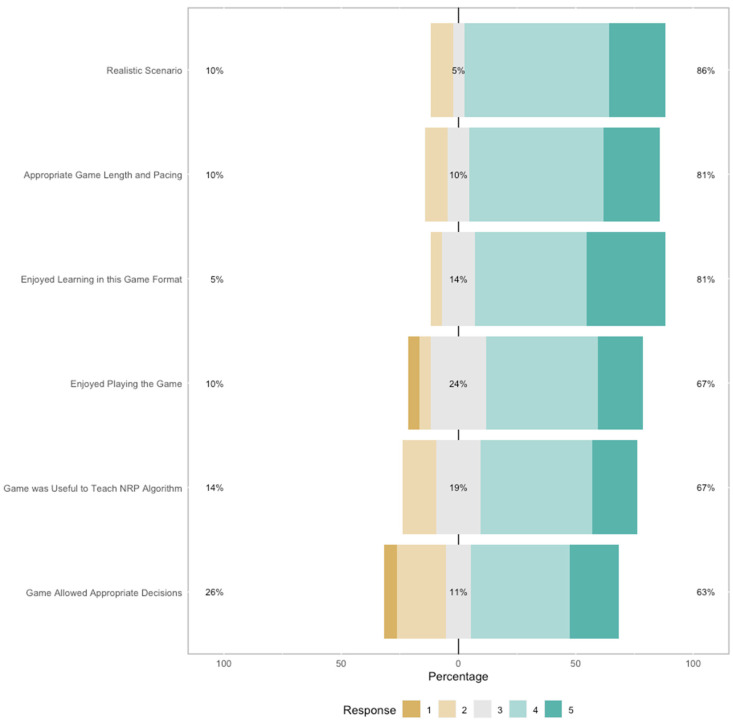
Participant responses on the game-related items included in the survey.

**Figure A1:**
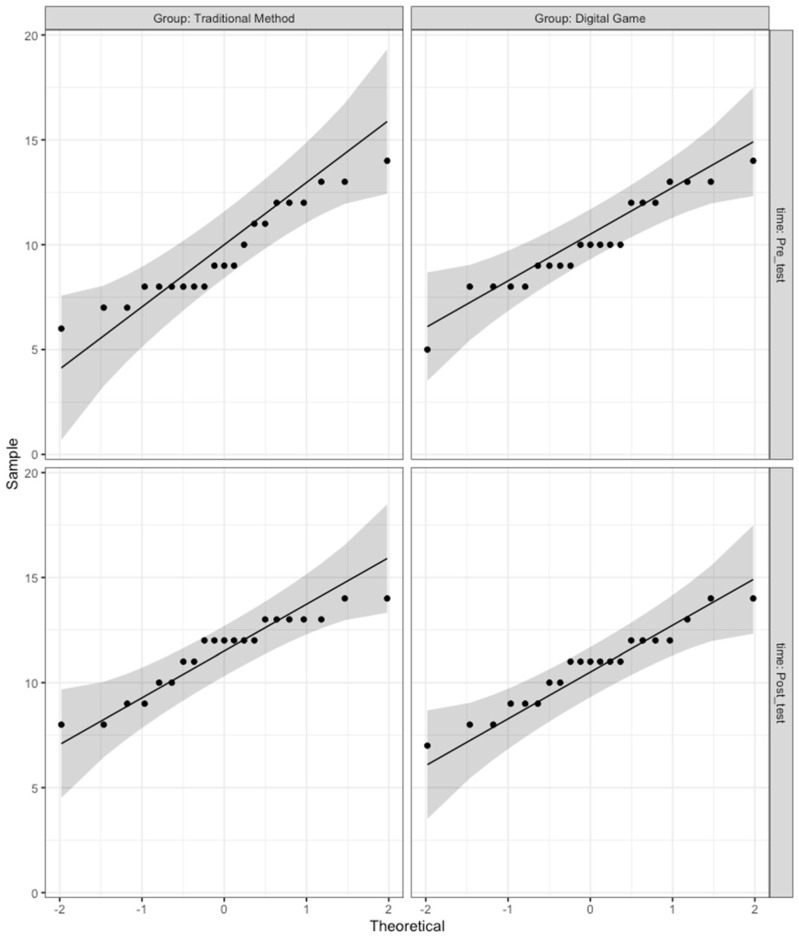
QQ plot of for each cell of the experimental design.

The findings shown in Table 6 and Figure 2 suggest that participants largely enjoyed playing the game, the game format of their training, and its usefulness to teach the NRP algorithm, thought that the neonatal resuscitation scenarios depicted in the game were realistic, and thought the pacing and game length were appropriate. To a smaller extent, they also thought that the game allowed them to make good decisions.

## 4. Discussion

### 4.1. Do the Two Instructional Groups Differentially Change Their Paramedics’ Neonatal Resuscitation Performance from a Pre-Test to a Post-Test?

The findings show that the performance of the participants on the neonatal resuscitation scenarios increased over time, but that the intervention (i.e., instructional method) did not make a difference in this increase. This indicates that, when the time at which the performance on the neonatal resuscitation scenarios was measured is ignored, the performance in the digital game group was not significantly different from the control group (i.e., traditional method). This result suggests that digital games are a viable training method whose effect on paramedics’ performances of neonatal resuscitation scenarios is not significantly different than that of more traditional training methods over time.

Moreover, participants can use digital games more readily than traditional methods to refresh their neonatal resuscitation knowledge, as these online games are more accessible than more traditional means of training. 

The results are consistent with similar findings in the literature. A recent literature review found that medical simulations were effective and motivating, providing a practical tool to help healthcare providers refresh their skills [22]. Some of the studies found no difference in performance measures between novel and traditional simulators. For instance, Curran et al. [23] found that completing an NRP tutorial using the ANAKIN simulator versus watching an NRP training video were as effective in maintaining the neonatal resuscitation skills of medical students. 

Overall, the results are encouraging, and they suggest that, with more iterations of development, RETAIN could one day surpass traditional methods, given that a recent meta-analytic comparative review showed that simulation-based medical education has been shown to be superior to the traditional approach to clinical teaching [24]. However, as research on simulation-based training methods is still relatively new, there are still mixed findings regarding their effectiveness compared to more traditional means, so more research in this direction is warranted.

### 4.2. Do Participants Perceive the Digital Game Positively?

The findings show that the paramedics largely enjoyed the game format and playing the game, as well as the affordances that the game offered for learning neonatal resuscitation knowledge and skills. This is not a surprising result, as digital games are by design equipped with several key characteristics that set them apart from other media [25]. This finding echoes the results of a different study revealing that medical students were satisfied with the simulator training [23]. Moreover, in contrast to standard neonatal simulations with a manikin, digital games include a non-linear trajectory that enable players to take on different identities and explore different ways of solving problems [12]. This level of autonomy and control is usually associated with more enjoyment [26,27]. 

Lastly, although only participants in the digital game group were asked whether they enjoyed the training environment, a recent review of simulation in neonatal resuscitation found that, in all studies, participants preferred to use high-fidelity simulators instead of the more traditional ones [28]. Taken together, the results suggest that participants find RETAIN to be an enjoyable digital game to play and also an enjoyable learning environment for neonatal resuscitation. This makes digital games like RETAIN an attractive alternative to refreshing one’s neonatal resuscitation knowledge.

### 4.3. Contributions of the Present Experimental Study

This experimental study makes several theoretical, methodological, and practical contributions to the neonatal resuscitation domain.

Theoretically, this randomized experimental study showed that digital games used to simulate neonatal resuscitation scenarios are able to capture paramedics’ neonatal resuscitation knowledge to a similar degree as traditional means of assessment. Thus, digital game simulators constitute a viable alternative to more traditional approaches that enable HCPs to keep their knowledge updated for solving neonatal resuscitation scenarios of varying difficulty. The findings also showed that playing the game was an enjoyable activity for most participants.

Methodologically, this study exemplifies a novel alternative to the traditional training of neonatal resuscitation skills, namely, a digital game that simulates neonatal resuscitation scenarios and that can be used whenever clinicians have spare time between their daily tasks. As the findings show that this new method provides similar results as traditional approaches, this simulation is a viable way of refreshing one’s neonatal resuscitation skills. It also provides a platform for more research and development in this area, which may improve HCPs’ outcomes. Moreover, experimentation with multimodal data and associated methodologies can be supported by digital game simulations like RETAIN. For instance, in a high-fidelity medical simulation, researchers employed think aloud protocols to capture the patient diagnosis process of experienced emergency physicians and residents [29]. Novel methods that blend qualitative with quantitative methodology are better suited for research in high-stakes environments where HCPs rely on applying accurately both their knowledge and creative strategies to solve complex and rarely occurring life-and-death problems.

Practically, this study also provides a practical and enjoyable way for HCPs to refresh or acquire knowledge on complex neonatal resuscitation scenarios. Many of the scenarios included in the game depict high acuity, low occurrence (HALO) events. Although very important, such HALO events may never occur in practice during the course of an HCP’s career. Therefore, digital games like RETAIN offer a safe platform for trying out various actions in response to complex scenarios and, thus, better prepare HCPs for real-life delivery room situations. In the future, more iterations and refinement of the RETAIN digital game may enable its use as a pedagogical training tool to either complement or replace standard training procedures.

### 4.4. Limitations and Future Work

This experimental study has several limitations.

First, the sample size was small, given the comparisons conducted across the two conditions. Please see Appendix A for the power analysis needed for this study. We strived to collect as much data as possible to enable conclusive results, but it is challenging to collect large samples from this population. Paramedics are very busy individuals, with an unpredictable schedule and limited time. They volunteered their free time to participate in this study during their breaks or outside their shift hours. As such, no new data collection is planned for this study. Despite the size of the sample, this experiment shows that digital games are promising in refreshing participants’ neonatal resuscitation knowledge, and that more research in this area is warranted to explore this instructional method.

Second, only participants who played the digital game were asked about their enjoyment of using that environment, due to time considerations. In future studies, data regarding enjoyment will be collected from participants learning in more traditional environments. 

Third, these results cannot be generalized to other populations, beyond paramedics. However, future studies will probe the generalizability of this instructional approach to other categories of healthcare providers to ascertain whether these results are stable across several healthcare provider populations.

## 5. Conclusions

This experimental study has sampled a population of paramedics and found that participants were able to improve their neonatal resuscitation performance from the pre-test to the post-test, but that this increase was not affected by the instructional method used (video lecture or digital game). This experiment has implications for the development of alternative methodologies and tools such as digital games that can be used in place of more traditional approaches of refreshing participants’ neonatal resuscitation performance, as the current results show no difference in participants’ increase in their neonatal resuscitation performance, due to the instructional method employed. Additionally, digital games like RETAIN have the advantage that they are enjoyable means of refreshing neonatal resuscitation performance.

## Figures and Tables

**Table 1:** Participant and study information.

Condition	Gender		Total
	Female	Male	
Traditional Method	9	12	21
Digital Game	5	16	21
Total	14	28	42

**Table 2:** Descriptive statistics of the variables included in this experiment.

Group	Pre-Test Mean (SD)	Post-Test Mean (SD)
Lecture Video (*n* = 21)	9.67 (2.31)	11.48 (1.83)
Digital Game (*n* = 21)	10.10 (2.23)	10.71 (1.90)

Note: SD = Standard Deviation.

**Table 3:** The results of the Shapiro–Wilk normality test.

Group	Pre-Test		Post-Test	
	Shapiro-Wilk	*p*	Shapiro-Wilk	*p*
Lecture Video (*n* = 21)	0.93	0.116	**0.91**	**0.049**
Digital Game (*n* = 21)	0.94	0.227	0.96	0.484

Note: The values marked in bold indicate statistically significant results.

**Table 4:** The results of the two-way mixed ANOVA.

Effect	*df*_1_	*df*_2_	*F(df*_1_ *, df*_2_ *)*	*p*	*η_p_* ^2^
Group	1	40	0.09	0.77	0.002
Time	1	40	**15.86** ***	<0.001	0.284 (large)
Group × Time	1	40	3.81	0.06	0.087

Note: *** *p* < 0.001

**Table 5:** Game-related 5-point Likert scale survey items included in the study (1 = Strongly Disagree, 2 = Disagree, 3 = Neutral, 4 = Agree, 5 = Strongly Agree).

Survey Item	Description
Realistic Scenario	The game scenario was realistic.
Appropriate Game Length and Pacing	The length of time and pacing during the game was appropriate to retain information of basic resuscitation steps.
Enjoyed Learning in this Game Format	I enjoyed learning neonatal resuscitation using this game format.
Enjoyed Playing the Game	I enjoyed playing the RETAIN game.
Game was Useful to Teach NRP Algorithm	This game is useful to teach the neonatal resuscitation algorithm.
Game Allowed Appropriate Decisions	The format of the game allowed me to make appropriate decisions.

**Table 6:** Descriptive statistics of the 5-point Likert scale game-related survey items.

Digital Game Group	*n*	Mean (SD)	Range
Realistic Scenario	21	4.00 (0.84)	2–5
Appropriate Game Length and Pacing	21	3.95 (0.87)	2–5
Enjoyed Learning in this Game Format	19	4.10 (0.83)	2–5
Enjoyed Playing the Game	21	3.71 (1.01)	1–5
Game was Useful to Teach NRP Algorithm	21	3.71 (0.96)	2–5
Game Allowed Appropriate Decisions	19	3.53 (1.22)	1–5

Note: SD = Standard Deviation.

## Data Availability

The data presented in this study are available on request from the corresponding author. The data are not publicly available due to privacy or ethical restrictions.

## Appendix A

As the post-test of the traditional method group was marginally normally distributed, a robust two-way mixed ANOVA was conducted using the bootstrap-based *bwtrim* function of the *WRS2* package [30] with 500 bootstrap samples and a modified one-step (MOM) estimator of location based on Huber’s Psi to fit between-within subjects ANOVA on the 20% trimmed means. The *WRS2* package also provides bootstrap-based functions for general M-estimators: *sppbb* for the within-subjects effect, *sppba* for the between-subjects effect, and *sppbi* for the interaction effect.

The *bwtrim* results of the two-way within-between subjects ANOVA showed that there was no interaction between time and condition predicting participants’ neonatal resuscitation performance, but that there was a statistically significant main effect of *time* on the neonatal resuscitation performance, as shown in Table A1.

**Table A1:** The results of the robust two-way mixed ANOVA using *bwtrim* with 20% trimmed means.

Effect	*df*_1_	*df*_2_	*F(df*_1_ *, df*_2_ *)*	*p*
Group	1	23.7865	0.13	0.72
Time	1	23.9825	**14.99** ***	<0.001
Group × Time	1	23.9825	3.74	0.06

Note: *** *p* < 0.001.

Although the analyses did not yield an interaction effect, given the main effect of time, follow-up tests (i.e., post-hoc comparisons) were performed on the single effect of time to determine whether any change in performance was simply due to time.

The *sppbb* results for the within-subjects effect (i.e., time) with an MOM estimator revealed a significant increase (estimate = −1.21, *p* = 0.008) from the pre-test to the post-tests on participants’ performance of neonatal resuscitation scenarios. The complete results of the robust two-way mixed ANOVA using the *WRS2* package are displayed in Table A2.

**Table A2:** The results of the robust two-way mixed ANOVA using sppbb, sppba, and sppbi.

Model	Effect	Estimate	*p*
*Sppbb*	Time (Pre–Post)	**−1.214** **	0.008
*Sppba*	Group (Traditional–Digital)	0.2568	0.738
*sppbi*	Interaction (Pre–Post × Traditional–Digital)	−0.8546	0.400

Note: ** *p* < 0.01. The bold font in the Estimate column indicates a statistically significant difference.

### ***Power Computations for Establishing the Required Sample Size***

It should be noted that we employed G*Power 3.1 to compute the sample size needed for a power of 0.80 in a 2 × 2 mixed design ANOVA. For the *F*-test family and the statistical test *ANOVA: Repeated measures, within-between interaction*, the estimation for a large partial eta squared effect size of *η_p_*^2^ = 0.25, with a correlation estimate of 0.5 between the repeated measures, a non-sphericity correction *ε* = 1, *α* = 0.05, and *β* = 0.20 (i.e., power = 0.80), yielded a sample of 10 participants, which is much less than the sample of this study.
